# FDG PET and MRI in Logopenic Primary Progressive Aphasia versus Dementia of the Alzheimer’s Type

**DOI:** 10.1371/journal.pone.0062471

**Published:** 2013-04-23

**Authors:** Ajay Madhavan, Jennifer L. Whitwell, Stephen D. Weigand, Joseph R. Duffy, Edythe A. Strand, Mary M. Machulda, Nirubol Tosakulwong, Matthew L. Senjem, Jeffrey L. Gunter, Val J. Lowe, Ronald C. Petersen, Clifford R. Jack, Keith A. Josephs

**Affiliations:** 1 Department of Radiology, Mayo Clinic, Rochester, Minnesota, United States of America; 2 Department of Health Sciences Research (Biostatistics), Mayo Clinic, Rochester, Minnesota, United States of America; 3 Department of Neurology (Speech Pathology), Mayo Clinic, Rochester, Minnesota, United States of America; 4 Department of Neurology (Behavioral Neurology), Mayo Clinic, Rochester, Minnesota, United States of America; 5 Department of Psychiatry and Psychology (Neuropsychology), Mayo Clinic, Rochester, Minnesota, United States of America; 6 Department of Information Technology, Mayo Clinic, Rochester, Minnesota, United States of America; Beijing Normal University, Beijing, China

## Abstract

**Objectives:**

The logopenic variant of primary progressive aphasia is an atypical clinical variant of Alzheimer’s disease which is typically characterized by left temporoparietal atrophy on magnetic resonance imaging and hypometabolism on F-18 fluorodeoxyglucose positron emission tomography. We aimed to characterize and compare patterns of atrophy and hypometabolism in logopenic primary progressive aphasia, and determine which brain regions and imaging modality best differentiates logopenic primary progressive aphasia from typical dementia of the Alzheimer’s type.

**Methods:**

A total of 27 logopenic primary progressive aphasia subjects underwent fluorodeoxyglucose positron emission tomography and volumetric magnetic resonance imaging. These subjects were matched to 27 controls and 27 subjects with dementia of the Alzheimer’s type. Patterns of atrophy and hypometabolism were assessed at the voxel and region-level using Statistical Parametric Mapping. Penalized logistic regression analysis was used to determine what combinations of regions best discriminate between groups.

**Results:**

Atrophy and hypometabolism was observed in lateral temporoparietal and medial parietal lobes, left greater than right, and left frontal lobe in the logopenic group. The logopenic group showed greater left inferior, middle and superior lateral temporal atrophy (inferior p = 0.02; middle p = 0.007, superior p = 0.002) and hypometabolism (inferior p = 0.006, middle p = 0.002, superior p = 0.001), and less right medial temporal atrophy (p = 0.02) and hypometabolism (p<0.001), and right posterior cingulate hypometabolism (p<0.001) than dementia of the Alzheimer’s type. An age-adjusted penalized logistic model incorporating atrophy and hypometabolism achieved excellent discrimination (area under the receiver operator characteristic curve = 0.89) between logopenic and dementia of the Alzheimer’s type subjects, with optimal discrimination achieved using right medial temporal and posterior cingulate hypometabolism, left inferior, middle and superior temporal hypometabolism, and left superior temporal volume.

**Conclusions:**

Patterns of atrophy and hypometabolism both differ between logopenic primary progressive aphasia and dementia of the Alzheimer’s type and both modalities provide excellent discrimination between groups.

## Introduction

Primary progressive aphasia (PPA) is a language disorder that is characterized by deficits in functions such as object naming, syntax, and word-processing [1]. There are three subtypes of PPA: the agrammatic, semantic, and logopenic variants [2]. In contrast to patients with the agrammatic and semantic variants, the majority of subjects with the logopenic variant of PPA (lvPPA) have been shown at autopsy or using amyloid-binding ligands such as Pittsburgh Compund B (PiB) [3] to have Alzheimer’s disease (AD) pathology [4]–[6]. Hence, lvPPA is considered an atypical presentation of AD. However, while typical dementia of the Alzheimer’s type (DAT) involves primarily loss of episodic memory, lvPPA is characterized by diminished single word retrieval and sentence repetition, and semantic and phonological paraphasias, with relatively preserved grammatical abilities, single-word comprehension and motor speech [2], [7].

Volumetric MRI has been studied in lvPPA and has shown characteristic patterns of grey matter atrophy and cortical thickness reduction in the left posterior temporal and inferior parietal lobes, with relative sparing of the sensorimotor cortex [8]–[11]. Similar and overlapping patterns of temporoparietal atrophy have been observed in DAT [9], [10]. Patterns of hypometabolism on F-18-fluorodeoxyglucose (FDG) PET have been reported in only a handful of lvPPA patients (n = 10), with similar patterns of left temporoparietal involvement observed [4], [12]. The degree of overlap in patterns of hypometabolism and atrophy within these syndromes, and between lvPPA and DAT, are however not fully understood. Comparing the structural and functional abnormalities of these two syndromes would help increase understanding of the mechanisms that drive heterogeneity in AD, and would be important for future studies evaluating environmental or genetic risk and monitoring progression in these syndromes.

The aims of our study were to examine the relationship between patterns of hypometabolism and grey matter atrophy in lvPPA and determine which combination of regions best differentiate lvPPA from DAT. In order to examine only subjects with AD pathology, we selected lvPPA and DAT subjects with amyloid deposition demonstrated on PiB-PET. We aimed to both contribute more statistical power to earlier findings by studying a larger number of lvPPA patients, and provide more comprehensive insight into the structural and functional deficits underlying lvPPA and DAT.

## Methods

### Subjects

We selected all subjects who had been recruited into a prospective study examining progressive speech and language-based disorders between November 29^th^ 2010 and July 20^th^ 2012 that met our clinical criteria for lvPPA. We selected only subjects who had a positive PiB-PET scan demonstrating the probable presence of AD pathology, since there is a suggestion that patterns of atrophy may differ between lvPPA subjects with and without AD pathology [13], [14]. A total of 27 subjects were identified. All subjects had undergone neurological evaluation by one Behavioral Neurologist (KAJ), detailed neuropsychological testing, and a detailed speech and language examination, as previously described [15]. Clinical diagnosis of lvPPA was rendered based solely on data from speech-language assessments without any reference to neurological or neuroimaging results. The diagnosis of lvPPA was independently determined by two speech-language pathologists (JRD and EAS); hence a consensus diagnosis. The clinical criteria used for lvPPA were as follows: 1) presence of aphasia, 2) impaired sentence repetition and comprehension, 3) presence of anomia with evidence of spared single word comprehension, 4) evidence of phonemic paraphasias, 5) normal rate of verbal expression or slowed verbal expression due to pauses for word retrieval without evidence of motoric slowing, and 6) absence of agrammatic/telegraphic verbal output. All 27 subjects also met recently published clinical diagnostic criteria for lvPPA [2].

The lvPPA subjects were matched 1∶1, as accurately as possible, to a cohort of 27 subjects with DAT on the basis of age and gender. Once again, we restricted our analysis to DAT subjects with positive PiB-PET scans. All DAT subjects were prospectively recruited into the Mayo Clinic Alzheimer’s Disease Research Center (ADRC) or Alzheimer’s Disease Patient Registry (ADPR) [16] between February 8^th^, 2010 and October 13^th^, 2011, and were identified from the ADRC/ADPR database. The diagnosis of DAT was made using established clinical criteria [17].

The lvPPA subjects were also matched 1∶1 to 27 cognitively normal controls based on age and gender. We only selected controls that were PiB-PET negative to avoid including subjects with preclinical AD. Controls were cognitively normal individuals that had been seen at Mayo Clinic for routine examinations and asked to enroll in the ADRC/ADPR. All controls were evaluated by a neurologist to ensure that they had normal neurological and neurocognitive examinations, and were not taking any medications that would affect cognition.

### Ethics Statement

This study was approved by the Mayo Clinic IRB. All subjects provided written informed consent before participating in any research activity. Because patients with receptive language impairment may have lost the meaning of words and may not be able to consent, assent was not sought. Capacity to consent was determined by the Behavioral Neurologist during clinical evaluation, utilizing a mental status examination. An acceptable legal authorized representative was considered only if that partner has power of attorney.

### Image Acquisition

MRI scans were performed with a standardized imaging protocol at 3T that included a 3D magnetization prepared rapid acquisition gradient echo (MPRAGE) sequence [18]. All MPRAGE images underwent pre-processing correction for gradient non-linearity [19] and intensity non-uniformity [20]. PET images were acquired after injection of C-11 PiB (average = 596 MBq; range = 292–729 MBq, uptake period = 40min) and F-18-FDG (average = 540 MBq; range = 366–399 MBq, uptake period = 30 min); both scans were performed as previously described on the same day with 1 hour between acquisitions [15].

### PiB-PET Analysis

A global cortical PiB retention summary was formed by calculating median uptake values in prefrontal, orbitofrontal, parietal, temporal, anterior cingulate, and posterior cingulate/precuneus regions and dividing this by median uptake in cerebellar gray matter. Subjects were classified as PiB-positive or negative using a global cortical-to-cerebellar ratio cut-point of 1.5 [21].

### Voxel-level Analyses

Voxel-level comparisons were performed for both MPRAGE and FDG-PET using SPM5 [22]. All MPRAGE scans were spatially normalized to a customized template [23] and segmented into gray matter (GM), white matter (WM) and cerebrospinal fluid using the unified segmentation model [24], followed by the hidden Markov random field clean up step [25]. All GM images were modulated and smoothed with an 8 mm full width-at-half maximum (FWHM) smoothing kernel. FDG uptake images were co-registered to the subject’s MPRAGE using 6 degrees-of-freedom registration. An in-house modified version of the automated anatomical labeling (AAL) atlas, containing pons, was propagated to native MPRAGE space and all voxels in the FDG-PET image were divided by median uptake of the pons to form FDG uptake ratio images. Native space GM and WM segmentations were then combined to form a brain tissue probability mask. The masks were re-sampled to the resolution of the PET images, smoothed at 6 mm FWHM, and used to perform a 2-compartment partial volume correction (PVC) [26] on the FDG uptake ratio images. Both the non-PVC corrected and the PVC corrected images were analyzed. The FDG images were then normalized to the customized template using the normalization parameters from the MPRAGE normalization and smoothed at 8 mm FWHM.

Voxel-level comparisons were performed between lvPPA and controls, DAT and controls, and lvPPA and DAT, using two-sided T-tests in SPM5. Differences between disease groups and controls were assessed at *p*<0.0005, after correction for multiple comparisons using the false discovery rate (FDR) correction, with an extent threshold of 100 voxels. Differences between lvPPA and DAT were assessed uncorrected for multiple comparisons at p<0.001 with an extent threshold of 100 voxels. Age and gender were included in all analyses as covariates, with total intracranial volume (TIV) included as an additional covariate in the GM comparisons, and time from onset to scan (disease duration) included as a covariate in the comparisons between lvPPA and DAT. Total intracranial volume was measured in SPM5 by propagating a template-drawn TIV mask to subject space, and then performing an erosion step to remove border voxels.

### Region-level Analyses

**Figure 1:**
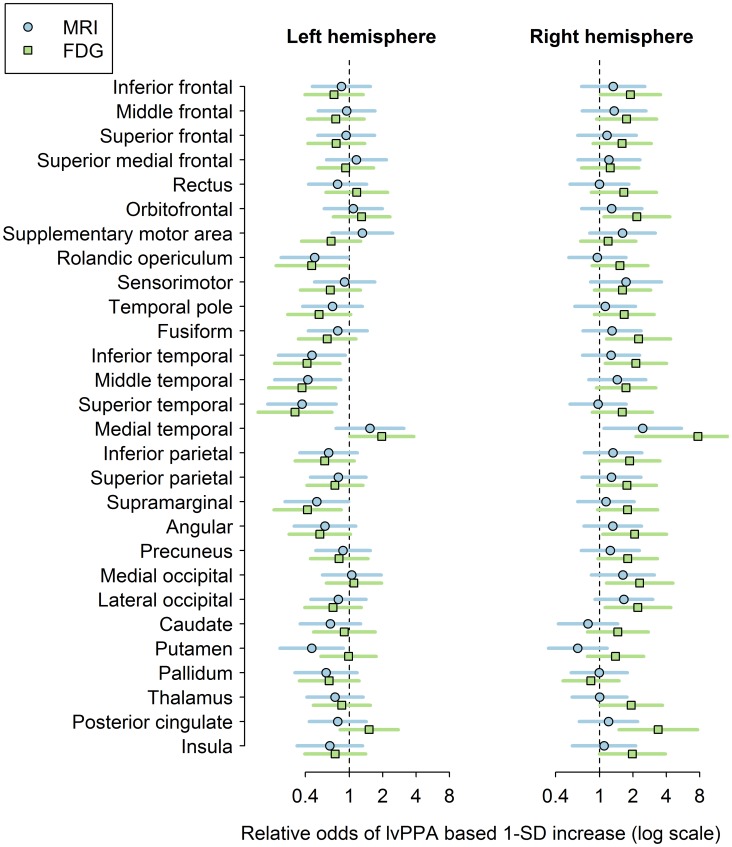
Region-level imaging comparison of lvPPA and DAT. Age-adjusted odds ratios and 95% CIs on the logarithm scale for a 1-SD change in the imaging variable. The vertical dashed line represents an odds ratio of 1.0. Estimates to the left of the vertical line indicate pathology tended to be greater in lvPPA. Estimates to the right of the vertical line indicate pathology tended to be less in lvPPA. 95% CIs that do not cross the dashed line represent significant differences between groups.

Atlas-based parcellation was employed using SPM5 and the in-house modified version of the AAL atlas [27], as previously described [28], in order to generate GM volumes and mean FDG uptake ratio for 28 regions-of-interest (ROI) that covered cortical and subcortical locations (see **Figure 1**). Left and right hemisphere values were assessed for all regions.

Briefly, the AAL atlas was transformed into native MPRAGE space. Binary native space GM probability masks were multiplied by the native-space AAL atlas, to generate a custom GM atlas for each patient, parcellated into the different ROIs. All regional GM volumes were divided by TIV to correct for differences in head size between subjects. The native-space custom GM+WM atlas for each subject was used to extract statistics from the FDG-PET images. Median values for each ROI were divided by the median value in the pontine ROI from the AAL atlas. Once again, we analyzed data with and without PVC.

### Statistical Analysis for Region-level Data

Demographic and cognitive variables were compared across all three groups using Kruskal-Wallis testing and host-hoc pairwise testing with Wilcoxon Rank Sum tests, or Chi-squared testing for categorical variables. Pair-wise group discrimination based on GM volume or FDG uptake ratios was assessed for each individual AAL-derived ROI using logistic regression models with age as a covariate and the imaging measure as the predictor.

A multivariable penalized logistic regression model was used in order to determine which combination of AAL-derived ROIs best discriminate lvPPA and DAT. All 28 ROIs were entered in the model to avoid the need for a priori hypotheses concerning which ROIs will be most useful for classification. The strength of this model is that it assesses the contribution of all ROIs to discrimination, and can determine whether each ROI adds additional information while controlling for the presence of the other ROIs. This approach ensures that potentially useful ROIs that may be somewhat unexpected would not be ignored. The penalized logistic regression model also protects against over-fitting given the relatively large number of predictors and small sample size. The amount of penalty was found through leave-one-out cross-validation [29]–[31]. To fit our multivariate models we used the glmnet software package and specified the tuning parameter alpha to be 80% so that the penalty was primarily constraining the sum of the absolute value of the coefficients which leads to a more parsimonious model with fewer ROIs: this still includes a constraint on the sum of the squared coefficients which provides more stability to the estimates when many are highly correlated [32]. We fit age- and disease duration-adjusted models based on (a) considering only GM variables, (b) considering only FDG variables, and (c) including variables from both GM and FDG ROIs. Using leave-one-out cross-validation we identified the penalty corresponding to a model that minimized error (defined as the change in model deviance) and then chose as our final model the one with the fewest number of ROIs but which had prediction error within 1 SE of the “optimal” model. This is a statistically conservative approach providing a somewhat more parsimonious model that is effectively equivalent to the “optimal” model in terms of model performance. To provide a summary of how well the models discriminate between lvPPA and DAT, we report the area under the receiver operating characteristic curve (AUROC) for the penalized logistic models. This statistic can be interpreted as an estimate of the rate of correctly classifying a randomly selected lvPPA and randomly selected DAT subject using the predictions from the model. We performed a bootstrap analysis based on replicating the cross-validation 1000 times to obtain approximate confidence intervals for the AUROC estimates and their differences. For univariate and multivariate modeling we standardize the predictors to have mean zero and SD 1 based using the mean and SD from the subjects in that particular analysis. All analyses were performed with R statistical software version 2.14.2 (R Foundation for Statistical Computing, Vienna Austria).

## Results

### Subject Demographics

By design, the groups did not differ in gender (**Table 1**). However, despite our best matching efforts, age differed across the groups, with the lvPPA group being the youngest (**Table 1**). Median global PiB ratios were lower in controls than lvPPA and DAT, but did not differ between lvPPA and DAT. Time from onset to scan differed between lvPPA and DAT. Performance on Mini-Mental State Examination, Boston Naming Test and Auditory Verbal Learning Test delayed recall differed across groups, with performance on Boston Naming Test significantly worse in lvPPA compared to DAT.

**Table 1:** Subject demographics.

	Controls (n = 27)	lvPPA (n = 27)	DAT (n = 27)	P values
Female, n (%)	14 (52)	14 (52)	13 (48)	0.95
Age, years	71±4[59–81]	65±10[47–85]	72±9[50–83]	0.005†
Time from disease onset to imaging, years	NA	3.4±1.5[1]–[6]	5.3±3.4[1]–[14]	0.01
Global PiB-PET ratio	1.3±0.1[1.2–1.4]	2.4±0.2[2.0–2.9]	2.4±0.3[1.8–3.1]	<0.001
Mini Mental State Exam score	28±2* [24]–[30]	21±6[10]–[30]	20±6* [6]–[29]	<0.001
Boston Naming Test (% correct)**	92±7[77–100]	45±30[0–100]	77±13[47–95]	<0.001§
Trail Making Test B	94±68[46–300]	246±98[36–300]	197±75[104–300]	<0.001
Auditory Verbal Learning Test delayed recall	7.8±3.3[0–14]	2.9±4.6[0–15]	0.9±2.3[0–10]	<0.001
Rey-Osterrieth complex figure test	NA	24±56[0–12.5]	22±11[2.5–30.5]	0.07

Data shown as mean ± standard deviation [range].

P values represent comparison across all three groups.

† lvPPA significantly different from DAT (p = 0.01).

§ All pair-wise comparisons were significant (p≤0.001).

* Short Test of Mental Status scores were converted to MMSE scores in the subjects recruited from the ADRC/ADPR using an algorithm developed at our center. [41].

** Boston Naming Test scores are shown as % of words correct out of total. DAT and control subjects received 60-item BNT and lvPPA subjects received 15-item BNT.

NA = Not available.

### lvPPA versus Controls

**Figure 2:**
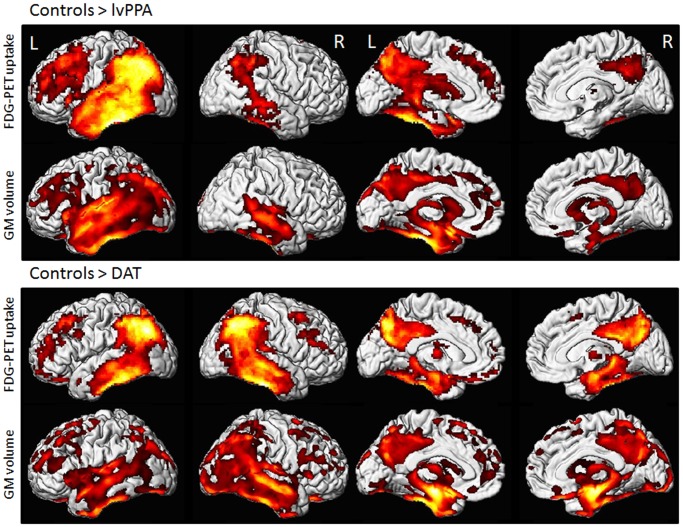
Voxel-level imaging findings in lvPPA and DAT when compared to controls. Three dimensional renderings show regions of reduced FDG metabolism and gray matter (GM) volume in lvPPA compared to controls and in DAT compared to controls. All images were generated using an FDR corrected statistical threshold of p<0.0005 and an extent threshold of 100 voxels. A decrease in brightness of the render reflects increased distance from the surface of the tissue.

The lvPPA group showed reduced metabolism and reduced grey matter volume in the same network of regions, involving left lateral temporal lobe, lateral and medial parietal lobes and frontal lobe, as well as right lateral temporal lobe and parietal lobe (**Figure 2**). Patterns of hypometabolism were less widespread after PVC, involving only left lateral temporal and inferior parietal lobes, posterior cingulate and precuneus. No regions showed greater abnormalities in controls compared to lvPPA.

### DAT versus Controls

The DAT group showed bilateral patterns of reduced metabolism and grey matter volume in medial and lateral temporal, parietal, and frontal lobes (**Figure 2**). Medial temporal findings included hippocampus, amygdala, and entorhinal cortex. Only regions of hypometabolism in lateral temporal lobe, lateral parietal lobe and posterior cingulate remained after PVC. No regions showed greater abnormalities in controls compared to DAT.

### lvPPA versus DAT

**Figure 3:**
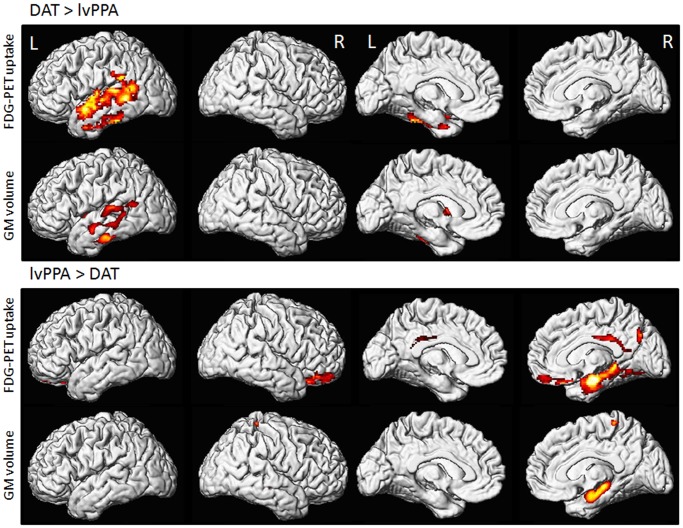
Voxel-level imaging comparison of lvPPA and DAT. Three dimensional renderings show regions of reduced FDG metabolism and gray matter (GM) volume in lvPPA compared to DAT, and in DAT compared to lvPPA. All images were generated using an uncorrected statistical threshold of p<0.001 and an extent threshold of 100 voxels. A decrease in brightness of the render reflects increased distance from the surface of the tissue.

In the voxel level analyses, lvPPA had greater hypometabolism and gray matter loss in left lateral temporal lobe, involving inferior, middle, and superior temporal gyri, than DAT (**Figure 3**). Conversely, DAT showed greater hypometabolism and more gray matter loss in right medial temporal lobe, particularly the hippocampus, compared to lvPPA (**Figure 3**). DAT also showed greater hypometabolism in right orbitofrontal lobe and posterior cingulate compared to lvPPA. Differences across groups remained significant after PVC.

Region-level differences between lvPPA and DAT are shown in **Figure 1** and **Table S1**. LvPPA showed both reduced metabolism and reduced volume in left inferior (p = 0.006, p = 0.02), middle (p = 0.002, p = 0.007), and superior temporal gyri (p = 0.001, p = 0.002), supramarginal gyrus (p = 0.008, p = 0.03), and rolandic operculum (p = 0.02, p = 0.03), compared to DAT. The left putamen also showed reduced volume in lvPPA compared to DAT (p = 0.01). Conversely, DAT showed reduced metabolism and volume in right medial temporal lobe (p<0.001, p = 0.02), and reduced metabolism in right posterior cingulate (p<0.001), occipital lobe (p = 0.01), angular gyrus (p = 0.02), inferior parietal (p = 0.04), fusiform gyrus (p = 0.01), inferior temporal lobe (p = 0.01), orbitofrontal cortex (p = 0.02), inferior frontal (p = 0.04), and thalamus (p = 0.04), and left medial temporal lobe (p = 0.04), compared to lvPPA. Similar FDG-PET findings were observed after PVC.

**Figure 4:**
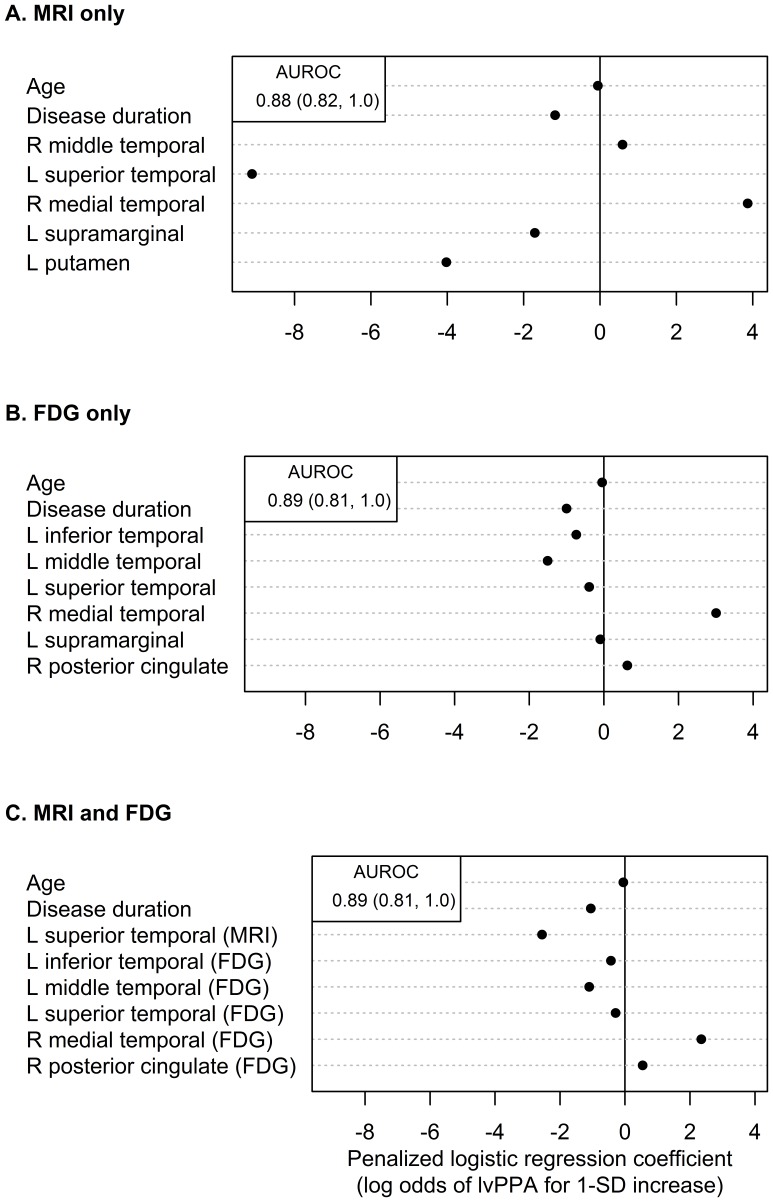
Estimated coefficients from multivariable penalized logistic regression analyses. Estimated AUROC and a 95% bootstrap confidence interval are shown in the top left of each panel.

These findings are substantiated by the penalized logistic regression models (**Figure 4**). The optimum model for MRI showed that excellent discrimination between lvPPA and DAT could be achieved (AUROC = 0.88) using right medial temporal lobe, right middle temporal gyrus, left superior temporal gyrus, left putamen, and left supramarginal gyrus. In contrast, the optimum model for FDG-PET (AUROC = 0.89) could be achieved using right medial temporal and posterior cingulate, and left inferior, middle and superior temporal lobes and left supramarginal gyrus. Finally, when FDG-PET and MRI were considered together, the optimum model (AUROC = 0.89) included mainly FDG-PET variables, involving all the variables highlighted in the FDG-PET only model, except the left supramarginal gyrus, as well as left superior temporal lobe volume. The AUROCs across all three models were not significantly different based on a bootstrap analysis.

## Discussion

We have described the imaging features of a relatively large cohort of lvPPA subjects with respect to FDG metabolism and gray matter volume. We found characteristic patterns of left-sided temporoparietal involvement in lvPPA, but also involvement of other regions in the language network, and showed that imaging variables can differentiate lvPPA from DAT.

With regard to the imaging characteristics of lvPPA, our results are consistent with previous findings in that we observed the most striking FDG hypometabolism and gray matter atrophy to be in the left lateral temporoparietal region [4], [8]–[12]. These regions have been shown to be associated with repetition deficits and phonological errors [33], [34], which are both features of lvPPA. However, we also saw involvement of left lateral and medial frontal lobe, right lateral temporal lobe, and left and right precuneus. These findings show involvement across almost the entire language network [35], suggesting that although pathology may begin in the left lateral temporal lobe, it then spreads throughout this network of regions. It is unsurprising that patterns of FDG-PET hypometabolism were very similar to patterns of atrophy, given that these measures are biologically related. Neuronal loss in the grey matter, measured as atrophy, will alter local neuronal connections and hence influence the synaptic activity measured indirectly by FDG-PET.

We also sought to understand how functional and structural anatomy in lvPPA compares to DAT. The left lateral temporal lobe was the main region where the imaging changes in lvPPA exceed that of DAT. This finding is consistent with the prominence of aphasia in lvPPA patients. It also concurs with the fact that cortical regions are typically more heavily involved on imaging and pathology in atypical AD compared to typical AD [11], [36]–[38], and that the distribution of neurofibrillary tangles is typically asymmetric in lvPPA, with greater involvement of the left hemisphere, yet symmetric in DAT [38]. Two previous studies similarly found greater left temporal atrophy in lvPPA compared to DAT [9], [11]. The FDG-PET analysis also showed greater hypometabolism in left inferior parietal regions in lvPPA than DAT, suggesting that FDG-PET may be more sensitive to differences in parietal regions. On-the-contrary, the main region where DAT showed greater imaging abnormalities than lvPPA was the medial temporal lobe. The medial temporal lobes were indeed relatively spared in the lvPPA subjects, yet are typically the primary focus of atrophy in DAT [11], [39]. This finding accounts for the fact that episodic memory impairment is typically more severe in DAT than lvPPA. The FDG-PET analysis also showed greater involvement of a number of parietal and occipital regions in the right hemisphere, particularly the posterior cingulate, in DAT compared to lvPPA, reflecting the fact that the right hemisphere is relatively spared in lvPPA.

Our multivariate logistic regression models were constructed to prevent over fitting and therefore be generalizable. The analysis revealed that both FDG-PET and MRI could differentiate lvPPA from DAT, with comparable accuracy. In both models the right medial temporal lobe (more involvement in DAT than lvPPA) and left lateral temporal regions (more involvement in lvPPA than DAT) were important for optimal discrimination. The right posterior cingulate was also a useful region in the FDG-PET models, while left putamen was useful in the MRI model. Both patterns of atrophy on MRI and hypometabolism on FDG-PET are therefore useful biomarkers to distinguish lvPPA from DAT, and only a few specific regions are necessary to aid diagnosis. Noticeably, in the model that included both modalities, the majority of the variables were FDG-PET variables, showing that FDG-PET is contributing more to optimum differentiation than MRI.

The strengths of our study include the relatively large cohort of lvPPA subjects and the inclusion of more than one imaging modality. Our lvPPA subjects were matched by age to the controls and DAT subjects, although young subjects are underrepresented in our ADRC/ADPR and, hence, there remained some age difference across groups. To address this concern we accounted for age differences in all our analyses. Our findings have important clinical ramifications. Diagnosing lvPPA clinically is a difficult task due to the complexity of the disease and the characteristics it shares with other forms of PPA and DAT. This diagnosis relies on cognitive-linguistic features of patients [40] which are variably present, often vary in severity, and sometimes co-exist with other cognitive-linguistic features. As the patterns of brain atrophy and hypometabolism in lvPPA are better characterized, we anticipate that imaging will become an increasingly important validating factor in lvPPA diagnosis. The specific imaging characterization of lvPPA that we have provided will be helpful in this regard. In addition, our results pertaining to the differentiation of lvPPA and DAT provide neuroanatomical explanations for the differing clinical presentations of these syndromes that share molecular pathology, and demonstrate that both MRI and FDG-PET can be useful clinically to help differentiate these syndromes. The differentiation of atypical variants of Alzheimer’s disease, such as lvPPA, from DAT could be very important for future clinical studies and treatment trials that utilize imaging biomarkers. Imaging biomarkers that are relevant as outcome measures in DAT will likely differ from those that are relevant in lvPPA. Identifying these patients before enrolment into a treatment trial will be critical.

## Supporting Information

Table S1 Regional grey matter volumes on MRI and FDG uptake in lvPPA and AD.
(DOCX)
