# Supplementary material for: FDG PET and MRI in Logopenic Primary Progressive Aphasia versus Dementia of the Alzheimer’s Type
Source: PLoS One. 2013 Apr 23;8(4):e62471. doi: 10.1371/journal.pone.0062471 (PMC3633885; doi:10.1371/journal.pone.0062471)
Supplement: Table S1 — Regional grey matter volumes on MRI and FDG uptake in lvPPA and AD. (DOCX) [file pone.0062471.s001.docx]

**Table S1**: Regional grey matter volumes on MRI and FDG uptake in lvPPA and AD.

|  | **MRI** | | | **FDG** | | |
| --- | --- | --- | --- | --- | --- | --- |
| **Region** | **lvPPA** | **AD** | **p*** | **lvPPA** | **AD** | **p*** |
| Inferior frontal |  |  |  |  |  |  |
| L | 0.78±0.10 | 0.77±0.10 | 0.58 | 1.39±0.14 | 1.42±0.15 | 0.29 |
| R | 0.86±0.12 | 0.81±0.11 | 0.39 | 1.56±0.12 | 1.46±0.17 | 0.04 |
| Middle frontal |  |  |  |  |  |  |
| L | 0.80±0.13 | 0.80±0.14 | 0.84 | 1.41±0.17 | 1.47±0.18 | 0.34 |
| R | 0.73±0.12 | 0.68±0.14 | 0.36 | 1.58±0.14 | 1.50±0.21 | 0.07 |
| Superior frontal |  |  |  |  |  |  |
| L | 0.50±0.09 | 0.49±0.10 | 0.82 | 1.34±0.14 | 1.39±0.17 | 0.35 |
| R | 0.55±0.08 | 0.53±0.11 | 0.62 | 1.48±0.11 | 1.42±0.17 | 0.12 |
| Superior medial frontal |  |  |  |  |  |  |
| L | 0.42±0.07 | 0.40±0.07 | 0.65 | 1.37±0.13 | 1.36±0.16 | 0.79 |
| R | 0.34±0.05 | 0.32±0.05 | 0.56 | 1.44±0.11 | 1.40±0.15 | 0.47 |
| Rectus |  |  |  |  |  |  |
| L | 0.17±0.02 | 0.17±0.03 | 0.42 | 1.41±0.12 | 1.37±0.13 | 0.64 |
| R | 0.17±0.02 | 0.16±0.02 | 0.99 | 1.46±0.12 | 1.38±0.13 | 0.13 |
| Orbitofrontal |  |  |  |  |  |  |
| L | 0.56±0.07 | 0.54±0.07 | 0.80 | 1.45±0.11 | 1.41±0.16 | 0.40 |
| R | 0.64±0.08 | 0.61±0.08 | 0.43 | 1.53±0.10 | 1.43±0.15 | 0.02 |
| Supplementary motor area |  |  |  |  |  |  |
| L | 0.26±0.04 | 0.24±0.04 | 0.40 | 1.47±0.12 | 1.52±0.13 | 0.21 |
| R | 0.28±0.04 | 0.25±0.05 | 0.15 | 1.53±0.10 | 1.51±0.12 | 0.54 |
| Rolandic opericulum |  |  |  |  |  |  |
| L | 0.20±0.03 | 0.21±0.03 | 0.03 | 1.33±0.16 | 1.41±0.12 | 0.02 |
| R | 0.27±0.04 | 0.26±0.04 | 0.87 | 1.48±0.13 | 1.43±0.12 | 0.15 |
| Sensorimotor |  |  |  |  |  |  |
| L | 1.04±0.20 | 1.01±0.20 | 0.75 | 1.45±0.12 | 1.49±0.10 | 0.20 |
| R | 1.04±0.17 | 0.92±0.20 | 0.13 | 1.54±0.09 | 1.50±0.10 | 0.10 |
| Temporal pole |  |  |  |  |  |  |
| L | 0.37±0.05 | 0.37±0.07 | 0.26 | 1.03±0.12 | 1.07±0.11 | 0.05 |
| R | 0.43±0.06 | 0.41±0.07 | 0.72 | 1.16±0.10 | 1.10±0.11 | 0.10 |
| Fusiform |  |  |  |  |  |  |
| L | 0.54±0.06 | 0.54±0.06 | 0.43 | 1.24±0.15 | 1.29±0.13 | 0.12 |
| R | 0.63±0.07 | 0.59±0.08 | 0.40 | 1.38±0.11 | 1.29±0.13 | 0.01 |
| Inferior temporal |  |  |  |  |  |  |
| L | 0.60±0.08 | 0.64±0.08 | 0.02 | 1.09±0.15 | 1.19±0.13 | 0.006 |
| R | 0.75±0.09 | 0.71±0.12 | 0.43 | 1.31±0.12 | 1.21±0.17 | 0.01 |
| Middle temporal |  |  |  |  |  |  |
| L | 0.72±0.08 | 0.79±0.09 | 0.007 | 1.08±0.16 | 1.24±0.17 | 0.002 |
| R | 0.82±0.08 | 0.79±0.12 | 0.22 | 1.32±0.13 | 1.26±0.20 | 0.07 |
| Superior temporal |  |  |  |  |  |  |
| L | 0.45±0.06 | 0.50±0.07 | 0.002 | 1.12±0.18 | 1.32±0.17 | 0.001 |
| R | 0.49±0.05 | 0.49±0.08 | 0.91 | 1.35±0.13 | 1.32±0.18 | 0.13 |
| Medial temporal |  |  |  |  |  |  |
| L | 0.50±0.05 | 0.47±0.07 | 0.23 | 1.11±0.09 | 1.04±0.10 | 0.04 |
| R | 0.53±0.04 | 0.47±0.08 | 0.02 | 1.17±0.06 | 1.04±0.11 | <0.001 |
| Inferior parietal |  |  |  |  |  |  |
| L | 0.33±0.06 | 0.35±0.08 | 0.14 | 1.17±0.22 | 1.29±0.18 | 0.09 |
| R | 0.19±0.03 | 0.18±0.05 | 0.35 | 1.39±0.21 | 1.30±0.21 | 0.04 |
| Superior parietal |  |  |  |  |  |  |
| L | 0.25±0.05 | 0.26±0.08 | 0.43 | 1.23±0.22 | 1.30±0.18 | 0.31 |
| R | 0.24±0.05 | 0.23±0.07 | 0.43 | 1.39±0.16 | 1.32±0.20 | 0.06 |
| Supramarginal |  |  |  |  |  |  |
| L | 0.24±0.03 | 0.25±0.04 | 0.03 | 1.15±0.18 | 1.31±0.17 | 0.008 |
| R | 0.31±0.04 | 0.30±0.05 | 0.66 | 1.36±0.16 | 1.30±0.18 | 0.06 |
| Angular |  |  |  |  |  |  |
| L | 0.19±0.03 | 0.20±0.04 | 0.10 | 1.11±0.18 | 1.24±0.19 | 0.05 |
| R | 0.31±0.04 | 0.31±0.06 | 0.37 | 1.35±0.21 | 1.26±0.24 | 0.02 |
| Precuneus |  |  |  |  |  |  |
| L | 0.52±0.08 | 0.53±0.09 | 0.65 | 1.34±0.22 | 1.41±0.18 | 0.48 |
| R | 0.57±0.07 | 0.55±0.10 | 0.47 | 1.46±0.18 | 1.39±0.19 | 0.06 |
| Medial occipital |  |  |  |  |  |  |
| L | 1.13±0.15 | 1.09±0.16 | 0.88 | 1.66±0.17 | 1.64±0.17 | 0.75 |
| R | 1.15±0.16 | 1.05±0.18 | 0.14 | 1.75±0.13 | 1.64±0.16 | 0.01 |
| Lateral occipital |  |  |  |  |  |  |
| L | 0.92±0.14 | 0.94±0.13 | 0.43 | 1.31±0.18 | 1.37±0.16 | 0.25 |
| R | 0.86±0.10 | 0.79±0.13 | 0.09 | 1.50±0.16 | 1.42±0.18 | 0.01 |
| Caudate |  |  |  |  |  |  |
| L | 0.19±0.03 | 0.19±0.03 | 0.21 | 1.35±0.16 | 1.32±0.15 | 0.74 |
| R | 0.21±0.03 | 0.21±0.03 | 0.44 | 1.35±0.16 | 1.27±0.13 | 0.24 |
| Putamen |  |  |  |  |  |  |
| L | 0.21±0.03 | 0.23±0.03 | 0.01 | 1.66±0.13 | 1.65±0.11 | 0.95 |
| R | 0.20±0.03 | 0.21±0.02 | 0.13 | 1.72±0.15 | 1.67±0.12 | 0.27 |
| Pallidum |  |  |  |  |  |  |
| L | 0.01±0.00 | 0.01±0.01 | 0.12 | 1.36±0.10 | 1.41±0.12 | 0.17 |
| R | 0.01±0.01 | 0.01±0.00 | 0.97 | 1.35±0.09 | 1.37±0.09 | 0.54 |
| Thalamus |  |  |  |  |  |  |
| L | 0.15±0.02 | 0.15±0.02 | 0.31 | 1.41±0.11 | 1.41±0.12 | 0.60 |
| R | 0.15±0.02 | 0.15±0.02 | >0.99 | 1.52±0.12 | 1.44±0.12 | 0.04 |
| Posterior cingulate |  |  |  |  |  |  |
| L | 0.06±0.01 | 0.06±0.01 | 0.42 | 1.38±0.20 | 1.31±0.18 | 0.17 |
| R | 0.03±0.00 | 0.03±0.01 | 0.55 | 1.51±0.20 | 1.34±0.18 | <0.001 |
| Insula |  |  |  |  |  |  |
| L | 0.38±0.06 | 0.38±0.06 | 0.23 | 1.30±0.10 | 1.30±0.12 | 0.35 |
| R | 0.39±0.06 | 0.36±0.08 | 0.79 | 1.38±0.10 | 1.30±0.13 | 0.04 |

Data is shown as mean ± standard deviation. Grey matter volumes are shown as a percentage of TIV. * Based on age-adjusted logistic regression model
